# Citizen science for healthier food environments: a realist-informed process evaluation of the big food map app in Flanders, Belgium

**DOI:** 10.3389/fpubh.2026.1742139

**Published:** 2026-03-10

**Authors:** Stefanie Vandevijvere, Jonathan Benhaiem, Lara Lamberts-Van Assche, Jessie Van Kerckhove, Suzannah D’Hooghe, Claire Maréchal, Myrthe Peijnenborg, Katrien Verbeke

**Affiliations:** 1 Sciensano, Brussels, Belgium; 2 Arteveldehogeschool, Ghent, Belgium; 3 Rikolto, Leuven, Belgium; 4 Let Us, Destelbergen, Belgium

**Keywords:** citizen science, co-creation, Flanders, food environments, food policy, health promotion

## Abstract

**Introduction:**

Food environments, the physical, economic, political, and sociocultural contexts shaping food availability, affordability, marketing, and social norms, strongly influence dietary behaviors and population health. The Big Food Map (*De Grote Voedselkaart*) was developed as a large scale citizen science initiative to engage citizens across Flanders, Belgium, in documenting and reflecting on their local food environments, aiming to raise awareness and support local action for healthy and sustainable food environments.

**Methods:**

The Big Food Map smartphone application was co-created with citizens, particularly adolescents and adults on lower incomes, as well as with actors, such as local governments, retailers, and short-chain initiatives. Co-creation involved Photovoice workshops with adolescents and adults on lower incomes and Group Model Building sessions with actors to explore how they understand food environments and identify key food environment dimensions and settings. Citizens and actors also tested and refined the app. The app was launched in May 2025 until August 2025 to crowdsource and provide feedback on citizen perceptions and behaviors related to neighborhood food environments. A brief evaluation questionnaire assessed the app’s usefulness and potential behavioral impact among citizens, while a more detailed survey explored stakeholder perspectives on the co-creation process and policy relevance.

**Results:**

In total 4,707 citizens contributed data across 1,320 neighborhoods (48% of all Flemish neighborhoods) and evaluated 11,313 food outlets. Participants appreciated the opportunity to visualize and discuss their local food environments. Approximately half liked the app’s design, 24% reported increased awareness of their food environment, and 56% said they would participate again. Both citizens and stakeholders highlighted challenges around long-term engagement, technical usability, and inclusion of vulnerable groups. The co-creation process strengthened partnerships between research, policy, and practice and underscored the value of tailoring citizen science tools to local contexts and communication channels.

**Discussion:**

The Big Food Map demonstrates the feasibility and potential of citizen science to enrich food environment monitoring and stimulate local dialog and action. To sustain engagement and policy relevance, future efforts should strengthen collaboration with local governments and media, diversify participation strategies, and embed citizen science tools into municipal food policy strategies or frameworks.

## Introduction

Unhealthy and unsustainable dietary patterns are major contributors to the global burden of noncommunicable diseases (NCDs) and environmental degradation (1, 2). These patterns are strongly shaped by the food environments in which people live (3, 4). Food environments encompass the physical, economic, political, and sociocultural contexts that determine what food is available, affordable, accessible, and desirable (5, 6). As such, they play a central role in shaping population health outcomes and in driving dietary inequalities within and across communities.

In recent years, improving food environments has become a major public health priority. International high level reports such as the WHO’s Report of the Commission on Ending Childhood Obesity (7) emphasize the need to transform food environments to make the healthy behavior the easy and preferred behavior in order to effectively address obesity and diet-related NCDs. Within the European Union, the Farm to Fork Strategy placed food system transformation at the core to creating healthier and more sustainable diets (8). In Belgium, and specifically in Flanders, food environments have recently also clearly been mentioned as an important concept within the umbrella food strategy, although specific policies and actions are still lacking (9). In Flanders, as in many high-income settings, citizens are increasingly exposed to ultra-processed foods, while access to affordable, healthy, and sustainable food options remains uneven (10–13).

Governments and civil society organizations are seeking practical ways to better understand and act on these food environment challenges (14). Traditional monitoring approaches, however, often only focus on objective food environments and lack the granularity and local ownership needed to drive community-level change (6, 15). Because food environments are inherently local, municipalities and community-level actors are particularly well positioned to identify challenges and implement tailored solutions (14, 16). Local actors however often lack a comprehensive and timely overview of subjective food environments, namely how their environments are perceived and experienced by different population groups, which limits their ability to design effective interventions and policies. Citizen science offers new opportunities to bridge this gap (17). By involving citizens directly in data collection, interpretation, and reflection, such approaches can democratize food environment research, enhance awareness, and strengthen the link between evidence and local action (18, 19). In addition, co-creation with diverse population groups, including adolescents and people in vulnerable situations, ensures that their perspectives and lived experiences are represented.

In food environment research, citizen science can capture subjective dimensions (20), such as perceived accessibility, affordability, or convenience, that are difficult to quantify in conventional audits. Combined with objective geospatial data, these perspectives offer a more holistic understanding of food environments and can inform targeted policy responses. Digital technologies, especially mobile applications, have expanded citizen science’s reach, enabling large-scale data collection, interactive feedback, and engagement of underrepresented groups (21). These tools can generate not only knowledge but also local dialog and collaboration among citizens, researchers, and policymakers.

This study presents a realist-informed process evaluation of the Big Food Map app (“De Grote Voedselkaart”), a citizen science initiative engaging citizens across Flanders to document and reflect on their neighborhood food environments. The process evaluation explored the contexts and mechanisms that shaped engagement, perceived usefulness, and potential policy impact. The study contributes to the evidence base on citizen-driven approaches to improving food environments and offers lessons for scaling and adaptation in other settings.

## Methods

Ethical approval for the co-creation activities with citizens and stakeholders was obtained from the Social-Societal Ethics Committee of KU Leuven (G-2023-7395). Approval for data collection through the app and evaluation questionnaires was granted by the Human Ethics Committee of Ghent University (ONZ-2025-0020). All participants provided informed consent, either written or via an online checkbox, before participating. The app did not require users to create personal accounts, ensuring minimal data collection and anonymity. No directly identifying personal data were collected.

The Big Food Map (“De Grote Voedselkaart”) was developed through a participatory citizen science initiative in Flanders, Belgium. The smartphone application integrated subjective data on perceptions and behaviors with objective geospatial data on local food environments. Co-creation involved adolescents, citizens on lower incomes, and multiple stakeholder groups, including local governments, retailers, and short-chain initiatives, to ensure inclusivity and relevance. The participatory approach sought to produce a tool that was practical for communities while also generating actionable data for local and regional policy actors. The process included four main stages: co-creation, app testing, data collection, and process evaluation.

### Co-creation

Evidence indicates that adolescents, and individuals from lower socioeconomic backgrounds are particularly vulnerable to obesogenic food environments (22, 23). To capture their perspectives on food environments and the most important dimensions and settings according to them, co-creation included two groups of adults on lower incomes from Ghent (*n* = 15) and three higher primary or secondary-school classes of adolescents in Antwerp (*n* = 11), Leuven (*n* = 16), and Ghent (*n* = 24), as well as actor groups of local governments (*n* = 12), retailers (*n* = 8), and short-chain initiatives (*n* = 8). Adolescents were recruited via schools, adults on lower incomes through community organizations (BMLIK, Kookploeg Solidair), and actors through partner institutions (Let Us, RIKOLTO, ILVO). The co-creation methods used were Photovoice with citizens and Group Model Building (GMB) with actors.

#### Photovoice

Photovoice was used to capture participants’ perceptions of their neighborhood food environments. This participatory method uses photography to elicit discussion and reflection on how environmental factors influence dietary behaviors either positively or negatively (24).

Over 2 weeks, participants photographed elements in their environments that encouraged or hindered healthy eating. Disposable or refurbished digital cameras were provided for participants without smartphones. Two sessions were held for each group: an introductory meeting to explain the process and a follow-up to discuss photographs and identify relevant themes related to food environments that facilitate or impede their eating behaviors. All photographs were printed for the discussions. Similar photos were collated. Based on the discussions with participants, and together with participants, researchers (LL and JV) coded relevant photos according to population group (adults on lower incomes, adolescents), type of food / setting (healthy vs. unhealthy; supermarket, school, social food provision, street, home, etc.), main influence or barrier (positive/negative feelings, facilitators, barriers), and key food environment dimensions (availability, affordability, accessibility, attractiveness, accommodation, acceptability, education, regulation, literacy) (25). Illustrative quotes or captions were extracted to convey participants’ lived experiences (e.g., “Cooking together builds community and supports food literacy, but limited resources hinder continuity”). Insights from photovoice guided the development of the food outlet review questions within the app.

#### Group model building

The group model building (GMB) technique was used within each group of actors (local governments, retailers, short chain initiatives) to explore the perceived drivers behind citizens’ healthy eating behaviors within food environments according to the actors (26). Actor groups (local governments, retailers, short-chain initiatives) identified factors influencing citizens’ dietary habits, which were then categorized by the researchers according to the food environment framework by Turner et al. (20). Causal loop diagrams (CLDs) were developed using Kumu to visualize relationships between factors and feedback loops shaping dietary patterns (27). Participants reviewed and validated the CLD diagrams. Insights from GMB informed both the app’s structure and the selection of perception and behavior questions.

### App testing and refinement

In total 10 citizens (adolescents and adults on lower incomes) and 18 stakeholders (representatives from local governments, retailers, short-chain initiatives, and project partner organizations) participated in iterative app testing rounds. Feedback was collected iteratively (e.g., three testing rounds). Feedback focused on usability, language, visual design, and functionality. Interviews among citizens and brief questionnaires among stakeholders captured experiences with software bugs, layout preferences, and navigation. Citizens and stakeholders also provided feedback on the branding, including the name, slogan and logo of the app/project. All questions and text contained in the app were reviewed by an organization (*De Kommaneuker*) that focused on clear simple communication toward a broad lay audience.

### Process evaluation

We described the profile of citizen participants using key user statistics derived from the full in-app dataset. Age (≥16 years) was summarized as mean (SD); gender, age group, dietary patterns, perceived income, education, and living situation were summarized as counts and percentages. Demographic questions were optional; missing responses are reported in the tables.

Engagement with the app was quantified using three activity components per participant: (1) weekly thematic challenges completed, (2) unique daily questions answered, and (3) location evaluations submitted. Accounts with identical creation and update timestamps and zero activity were labeled non-active.

To contextualize engagement over time and by outreach channel, we compiled communication campaign analytics, including impressions, click-through rate, and cost per click for online newspaper adverts and Meta adverts, as well as post-level metrics on HOPLR (views, reactions, comments). Time-series plots of daily submissions were compared with the timing of major communication pushes to assess alignment between outreach and participation peaks.

Two short evaluation questionnaires were administered: one for citizen participants and one for stakeholder organizations. Both combined closed-ended Likert items with optional open-ended questions. The citizen survey assessed (i) clarity of project goals and expectations, (ii) perceived design and usability (e.g., visual appeal, ease of navigation), and (iii) perceived impact (awareness of the local food environment and of one’s dietary behaviors; perceived likelihood of local action). Distribution occurred via three channels to maximize reach: within the app, a project newsletter, and the HOPLR social network. Responses were summarized descriptively; agreement was reported as the proportion selecting “agree” or “totally agree.” The stakeholder survey targeted project partners. It covered (i) co-creation processes (inclusiveness, usefulness, legitimacy), (ii) design/usability feedback, (iii) perceived policy relevance, and (iv) suggestions for sustaining engagement and improving communication. Items used 4- or 5-point Likert scales; we report median scores and IQRs given small group sizes. Free-text comments were inductively coded to identify common themes (e.g., user-friendliness, visibility, inclusion of vulnerable groups).

To evaluate engagement and perceived impact, the process was guided by a Context–Mechanism–Outcome (CMO) framework derived from realist evaluation (28). This approach is well suited to participatory interventions, allowing exploration of how and why outcomes occur, for whom, and under what circumstances. In line with earlier applications in public health and participatory research (29), we conceptualized context as the social, institutional, and technical conditions shaping the development and uptake of the app; mechanisms as the engagement processes such as ownership, empowerment, legitimacy; and outcomes as the intended and unintended changes in awareness, collaboration and local food environment actions. Data sources included app analytics, citizen questionnaires, stakeholder surveys, and communication campaign metrics, which were triangulated for interpretation.

## Results

### Citizens’ perspectives on their local food environments

The key dimensions in food environments according to adults on lower incomes (*N* = 15) and adolescents (*N* = 51) through photovoice studies are summarized in Table 1. A more elaborated summary can be found in Supplementary material S1.

**Table 1:** Key dimensions in food environments according to adults on lower incomes (*N* = 15) and adolescents (*N* = 51) through photovoice studies.

Group	Setting/food type(s)	Main influence (feelings and drivers)	Key dimensions food environment	Illustrative insight
Adults on lower incomes	Social food provision/healthy foods	Hopeful: social connection, informal literacy. Barriers: lack of time, staff, resources	Attractiveness, affordability, accommodation, literacy	Cooking together builds community and supports food literacy, but structural barriers limit sustainability
	Fast-food outlets/unhealthy foods	Negative: unhealthy food highly visible and accessible; healthy outlets less visible	Attractiveness, accessibility, accommodation	Urban planning and retail practices promote unhealthy food behaviors
	Supermarket promotions/mixed (healthy and unhealthy foods)	Positive: affordable promos for healthy foods help avoid behavior of processed foods	Attractiveness, price, affordability	Promotions on healthy foods act as facilitator for healthy diets
Adolescents	Kebab/French fries near school/mixed (healthy and unhealthy foods)	Highly attractive, linked to taste and emotions; request-driven	Attractiveness, availability, accessibility	Marketing and proximity drive frequent requests for fast food
	Supermarket promotions and packaging/mixed (healthy and unhealthy foods)	Promotions motivate pester power; packaging (cartoons) drives demand	Attractiveness, affordability, availability	Marketing strongly shapes dietary behaviors; cartoons could work for healthy foods
	Vegetables at home/healthy foods	Acceptance depends on preparation and texture	Acceptability	Children prefer raw/crunchy over cooked vegetables
	Eating out with friends (kebab, restaurants)/unhealthy foods	Social norms and group practices strongly drive behaviors	Attractiveness (social), acceptability	Peer culture reinforces eating out, often unhealthy dietary behaviors
	School vending machines/mixed (healthy and unhealthy foods)	More use of healthy options when cheaper and more prominently placed	Accessibility, price	Placement and pricing can shift dietary behaviors toward healthy
	Supermarkets/mixed (healthy and unhealthy foods)	Lidl favored for affordability; Delhaize favored for quality but too expensive	Price > acceptability, availability	Price is the dominant driver in dietary behaviors within food outlets

Through the Photovoice activities, participants captured and discussed a wide variety of environmental influences on their diets. Photographs often depicted the perceived dominance of fast-food outlets, sugar-sweetened beverages, and promotional materials in public spaces. Across both adolescents and adults on lower incomes, unhealthy food options were perceived as more visible, accessible, and attractive than healthy options. Participants described being “tempted” by visual and sensory cues such as smell, color, and packaging, while healthy foods were viewed as less convenient, less visible, or less appealing.

Affordability emerged as a recurrent concern, particularly among adults on lower incomes, who emphasized that the high price of fresh produce and healthy meals, combined with limited mobility or time, constrained healthier choices. Social food initiatives such as social restaurants, food banks, and community kitchens were valued not only for affordability but also for fostering social connection and food literacy. However, participants pointed to differences in accessibility, quality, and coordination between such services, as well as a lack of visibility or clear information about eligibility.

Among adolescents, marketing, peer influence, social norms, and family habits emerged as powerful mechanisms shaping preferences. Bright packaging, cartoon characters, and “2-for-1” promotions encouraged them to request or choose specific products, often high in sugar, salt, or saturated fat. Advertising—especially for fast food—was described as omnipresent and effective in creating cravings, while healthy foods rarely received similar exposure. Nonetheless, adolescents recognized that better presentation, variety, and taste of healthy options (e.g., colorful fruit salads instead of plain fruit) could increase their appeal. They also valued trust and familiarity in food sources—preferring known supermarkets or restaurants—and saw schools as important settings to make healthy options more attractive, available, and affordable (Table 1; Supplementary material S1).

### Key actors’ perspectives on drivers of dietary behaviors

The main factors influencing dietary habits of citizens according to key actors within different food environment dimensions are presented in Table 2.

**Table 2:** The main factors influencing dietary habits of citizens according to key actor groups within different food environment dimensions.

Domain	Dimension	Local governments	Short chain initiatives	Retailers (primarily supermarkets)
External food environment	Availability	- Availability of healthy foods - Availability of unhealthy foods - Degree of autonomy in food behaviors in different settings (i.e., number/variety of behaviors in nursing homes, hospitals, etc.) - Freedom of choice - Unhealthy food options for children in restaurants - Variety of food options in the work/school cafeteria - Availability of inexpensive food options	- Availability of healthy foods - Availability of unhealthy foods	- Availability of healthy food outlets - Availability of unhealthy food outlets - Availability of healthy foods - Availability of unhealthy foods - Degree of autonomy in food behaviors in different settings (i.e., number/variety of behaviors in nursing homes, hospitals, etc.) - Availability of fresh produce (fruits and vegetables) - Availability of healthy foods for vulnerable groups - Variation in food options - Seasonal variation in food options
	Prices	- Price of healthy foods - Price of unhealthy foods - Price promotions (1 + 1; % reduction)		- Price of healthy foods - Price of unhealthy foods - Price promotions (1 + 1; % reduction) - Price promotions for big brands - Price/quality for healthy foods
	Vendor and product properties	- Opening hours of points of sale - Options for delivery - Supermarket layout - Shelf space/height - One-stop shop - Music - Cozy décor/lay-out - Child-friendly environment - Candy at the checkout - Health-related labeling on foods - Culturally diverse offer - Variety in package/serving sizes - Seasonal food	- Opening hours of points of sale - Cozy décor/lay-out - Shopping experience - Nutritional quality of foods - Health-related labeling on foods - Shelf life - Portion sizes of foods - Freshness of foods	- Opening hours of points of sale - Options for delivery - Payment methods - Health-related labeling on foods - Nutritional quality of foods - Shelf life - Nutritional quality of foods - Information at point of purchase - Transparency (i.e., claims)
	Marketing	- Marketing for unhealthy foods - Marketing for healthy foods	- Marketing for unhealthy foods	- Marketing for unhealthy foods - Marketing for healthy foods
	Regulation	- Nutri-Score - Rules/regulations/standards at school/at work	- Nutri-Score - Preventive healthcare - Extent of regulation	
	Education	- Education (i.e., recipe books) - News about scarcity/price increases	- Education about quality and origin of foods - Misinformation about food - Campaigns by government	
Personal food environment	Accessibility	- Distance to food offer - Access to public transportation - Accessibility of supermarket - Parking space	- Distance to food offer - Access to public transportation - Access by bicycle/on foot - Access by car - Accessibility of healthy foods - Distance to healthy foods - Distance to supermarkets	
	Affordability	- Prioritizing having food over eating healthy	- Perception affordability of healthy foods - Food as a financial priority	
	Convenience	- Convenience	- Convenience - Time to shop/cook/eat - Convenience of fast food - Convenience of supermarkets - Convenience of food box/one-stop shop	
	Acceptability	- Culturally appropriate food options - Social/peer pressure from children related to unhealthy foods - Diversity in packaging/serve sizes	- Eating culture - Eagerness to cook - Demand healthy foods - Priority healthy eating - Attention to farmers’ wellbeing - Addictiveness unhealthy foods - Food loss and waste	- Demand healthy foods
	Desirability	- Attractiveness of packaging - Promotions on healthy food prices - Prominence of unhealthy foods - Taste - Promotions on unhealthy foods (1 + 1; while stocks last) - Promotions on large packages - Smell - Candy at the checkout - Samples for tasting - Healthy eating campaigns - Healthy eating challenges - Social pressure to eat healthy - Social norms - Influencers - Cooking programs - Marketing through celebrities	- Taste - Personalized digital marketing - In-store marketing - Positive modeling (through teachers, parents, peers) - Negative modeling (through teachers, parents, peers) - Normalization of healthy eating - Individualism - Community feeling - Transparency short chains - Attention to farmers’ wellbeing	- Taste - Attention to origin of foods - Attention to local foods - Attention to production methods of foods
Other personal factors	Other personal factors	- Skills related to healthy eating - Nutrition literacy - Number of children in the household - Network	- Cooking skills - Behavior motives: environment, sustainability - Moral behavior motives (animal welfare) - Awareness healthy eating - Confidence in the food system	- Mental space - Sensitivity to addiction - Infrastructure (home, kitchen) - Income

**Figure 1:**
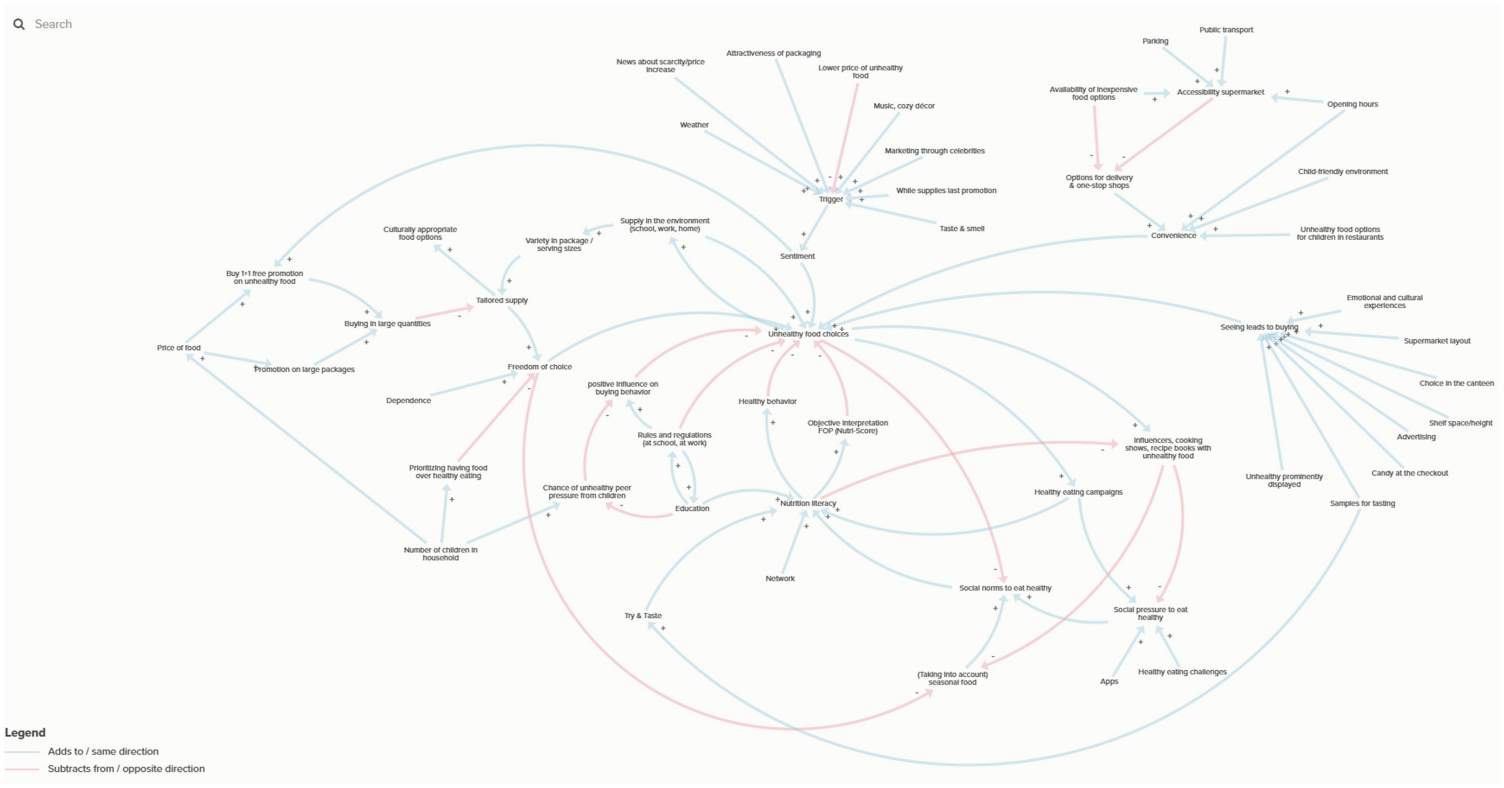
CLD developed by local government actor group. An interactive version of the figure is available at: https://embed.kumu.io/ebabe5a690c3594e47ca1fdc51681dcb#testeng, licensed under CC BY-SA 3.0.

Across actor groups (local governments, short chain initiatives, and retailers), a wide range of factors were identified as shaping citizens’ dietary habits through the Group Model Building technique. Each group collaboratively developed a causal loop diagram (CLD) representing factors they considered influential. The primary purpose of these workshops was to elicit and structure relevant variables, which are summarized in Table 2. While the resulting CLDs served mainly as a participatory exercise to surface and discuss these variables, they also provided insight into the complexity of relationships among them. Figure 1 presents one illustrative CLD of the actor group with stakeholders from local governments, highlighting how the diverse variables identified by participants are interlinked and may shape dietary behaviors.

In the external food environment, actors emphasized both the availability of healthy and unhealthy foods and outlets, the price of these foods (including promotions), and vendor characteristics such as opening hours, supermarket layout, labeling, and product variety. Marketing was consistently noted as a driver, with a predominance of unhealthy food marketing but also some recognition of the potential of healthy food promotion. Regulation (e.g., Nutri-Score, school or workplace standards) and education campaigns were seen as further levers. In the personal food environment, accessibility (distance, transport, supermarket access), affordability perceptions, and convenience of shopping or eating were mentioned as key influences. Acceptability was linked to culturally appropriate foods, peer and social norms, and cooking practices, while desirability was strongly shaped by taste, packaging, promotions, in-store marketing, media representations, and social modeling. Finally, actors pointed to other personal factors such as cooking skills, nutrition literacy, income, and broader moral or sustainability motives as additional determinants. Together, these findings highlight the multifaceted nature of food environments and the diverse entry points for local action.

Local governments emphasized structural issues such as zoning regulations, density of fast-food outlets, and limited resources for healthy-eating promotion. They saw potential for community-level data from the app to inform urban-planning and health initiatives. Retailers recognized their influence through product placement, pricing, and in-store marketing, but noted challenges balancing commercial objectives with health promotion. Short-chain initiatives (e.g., local producers, farmers’ markets) viewed the app as an opportunity to increase visibility and connect with consumers interested in sustainability.

### App content and design

The co-creation and testing processes led to the final structure, appearance, and functionality of the Big Food Map app. Stakeholder and citizen feedback guided every stage of design.

#### Food environment map and data sources

Food selling points were collected and added on the map of Flanders from several sources:

- Dataset containing all food retailers and restaurants in Flanders for the year 2024 as derived from Locatus (http://www.locatus.com) ©. The geographical coordinates, name and address, retail type and floor size of each individual outlet are included in the database. The frequency of field audits varies from once a year—in shopping centers—to once every 2 or 3 years in locations outside of shopping centers. The Locatus database was field validated in the Netherlands and was shown to be highly accurate (30).
- Database that contains diverse types of short-chain initiatives in Flanders as derived from Recht van bij de boer (http://www.rechtvanbijdeboer.be) ©.
- Database that contains social food facilities and provisions, such as social restaurants, food banks, distribution platforms, social grocery stores, in Flanders as derived from de Sociale kaart (http://www.desocialekaart.be)
- Database containing all primary and secondary schools as well as universities and university college campuses in Flanders
- Database containing all sport and recreation centers in Flanders

All food selling points were divided into 8 categories (supermarkets; fast food and takeaway; full-service restaurants; short-chain initiatives; convenience stores; schools; sport and leisure centers; social food facilities). Based on prior research (11), a map of the food environment of Flanders was created containing the food health scores of all neighborhoods. An expert committee in Flanders consisting of 15 dieticians, food scientists and food policy advisors categorized each food retailer type according to healthiness on a Likert scale from 1 to 5. These ratings were used to compute an average neighborhood-level food health score, color-coded from red (least healthy) to green (most healthy).

#### Citizen tasks and feedback

The questions and tasks for citizen scientists were divided in three categories. A complete overview can be found in Supplementary material S2.

- Evaluation of food selling points: User could evaluate relevant selling points by clicking on the point on the map, and answering a set of questions that were randomly selected from a list of 10 questions, specifically designed per food selling point category.
- Answering daily questions: Users were asked about their behaviors and perceptions in the food environment. Each question could only be answered by selecting a specific food selling location as the response.
- Completing weekly thematic challenges: Users were invited to complete thematic challenges once per week which contained a combination of photo tasks, open field questions, multiple behavior and slider-rating questions. Several themes were explored across the eight-week campaign, such as food advertising, short-chain initiatives, ideal food environments, social norms, and eating habits.

Citizens received direct feedback on their responses. For each multiple-behavior question, participants were shown how their answers compared to those of other respondents in the user base. This feedback was provided only for multiple-behavior items.

#### Visual identity and branding

The design, branding, concepts were created in a process of co-creation between the app developers relevant stakeholders. An overview of design and branding is presented in Supplementary material S3.

o Name: “The Big Food Map” (“De Grote Voedselkaart”)

The name of the project was carefully constructed and considered. ‘Big’ refers to the scale of the project; it involves the entirety of Flanders. ‘Food’ is the main subject of the project. More precisely, the food environment of Flanders. Map, because the map is the main tool of the project, and takes the center stage in the app.

o Slogan “Exploring the food-neighborhood together” (“Samen de voedselbuurt verkennen”)

The slogan was constructed to engage potential participants. ‘Exploring’ evokes feelings of researching, and aims to stimulate the citizen to participate. ‘Food-neighborhood’ focusses the attention on the location where the research will happen. Citizens will explore their own food-neighborhood. ‘Together’ alludes to the collective action and consciousness, stimulating citizens to collaborate on a local level for regional change.

o Logo, branding

The logo is constructed from the name, the slogan, and three visual elements that relate to the food environment: (1) carrot (healthy food), (2) shopping basket (food environment), (3) magnifying glass (research).

o App design/flow

The app aimed to combine objective and perceived data to stimulate reflection and discussion on local food environments. The project was to be playful yet informative, direct and clear, activating, and casual. All these elements were reflected in the final product design and branding (Supplementary material S3).

### User engagement and reach

Between May and August 2025, 4,707 users contributed data across 1,320 neighborhoods (48% of total neighborhoods in Flanders). In total, 11,313 food outlet evaluations were collected, covering 5,036 unique venues (an average of two reviews per outlet). The largest share of evaluations concerned supermarkets (4,927; 44%), followed by restaurants (1,861; 16%), fast-food and take-away outlets (1,743; 15%), local shops (1,451; 13%), and short food supply chain outlets (1,039; 9%). A smaller number of reviews were recorded for recreational facilities (150; 1%), educational settings (106, <1%), and social food initiatives (36, <1%). Altogether, these represent evaluations for about 8% of all 63,848 food outlets in Flanders.

Participants were mostly women (70%), and middle-aged adults (mean age 51). About 13% of participants reported difficulties to make ends meet (Table 3).

**Table 3:** Participant demographics (All participants; Active participants; % include missing).

	All participants	Active participants
Characteristic	*N* = 4,707	*N* = 2,364
Age, Mean (SD)	50.8 (14.7)	47.9 (14.1)
Missing/prefer not to say	8	2
Gender, *n* (%)		
Female	3,252 (69.9%)	1,706 (72.9%)
Male	1,399 (30.1%)	633 (27.1%)
Missing/prefer not to say	56	25
Diet, *n* (%)		
Omnivore	2,666 (59.0%)	1,318 (56.8%)
Flexitarian	973 (21.5%)	523 (22.6%)
Vegetarian	242 (5.4%)	140 (6.0%)
Vegan	112 (2.5%)	76 (3.3%)
Pescatarian	126 (2.8%)	70 (3.0%)
Medical dietary restrictions	344 (7.6%)	176 (7.6%)
Religious dietary restrictions	54 (1.2%)	16 (0.7%)
Missing/prefer not to say	190	45
Perceived income, *n* (%)		
Very difficult	135 (2.9%)	55 (2.4%)
Difficult	457 (10.0%)	210 (9.0%)
Neither difficult nor easy	1,573 (34.3%)	729 (31.4%)
Easy	1,688 (36.8%)	918 (39.6%)
Very easy	736 (16.0%)	409 (17.6%)
Missing/prefer not to say	118	43
Education, *n* (%)		
No diploma	72 (1.6%)	21 (0.9%)
Primary education	71 (1.5%)	28 (1.2%)
Secondary education	904 (19.5%)	366 (15.7%)
Post-secondary vocational	347 (7.5%)	161 (6.9%)
Higher/university education	3,238 (69.9%)	1,760 (75.3%)
Missing/prefer not to say	75	28
Living situation, *n* (%)		
Cohousing	110 (2.4%)	66 (2.8%)
Living alone	901 (19.5%)	418 (17.9%)
With children, no partner	284 (6.1%)	153 (6.5%)
With parents	111 (2.4%)	57 (2.4%)
With partner and children	1,521 (32.9%)	838 (35.9%)
With partner, no children	1,699 (36.7%)	805 (34.4%)
Missing/prefer not to say	81	27

**Figure 2:**
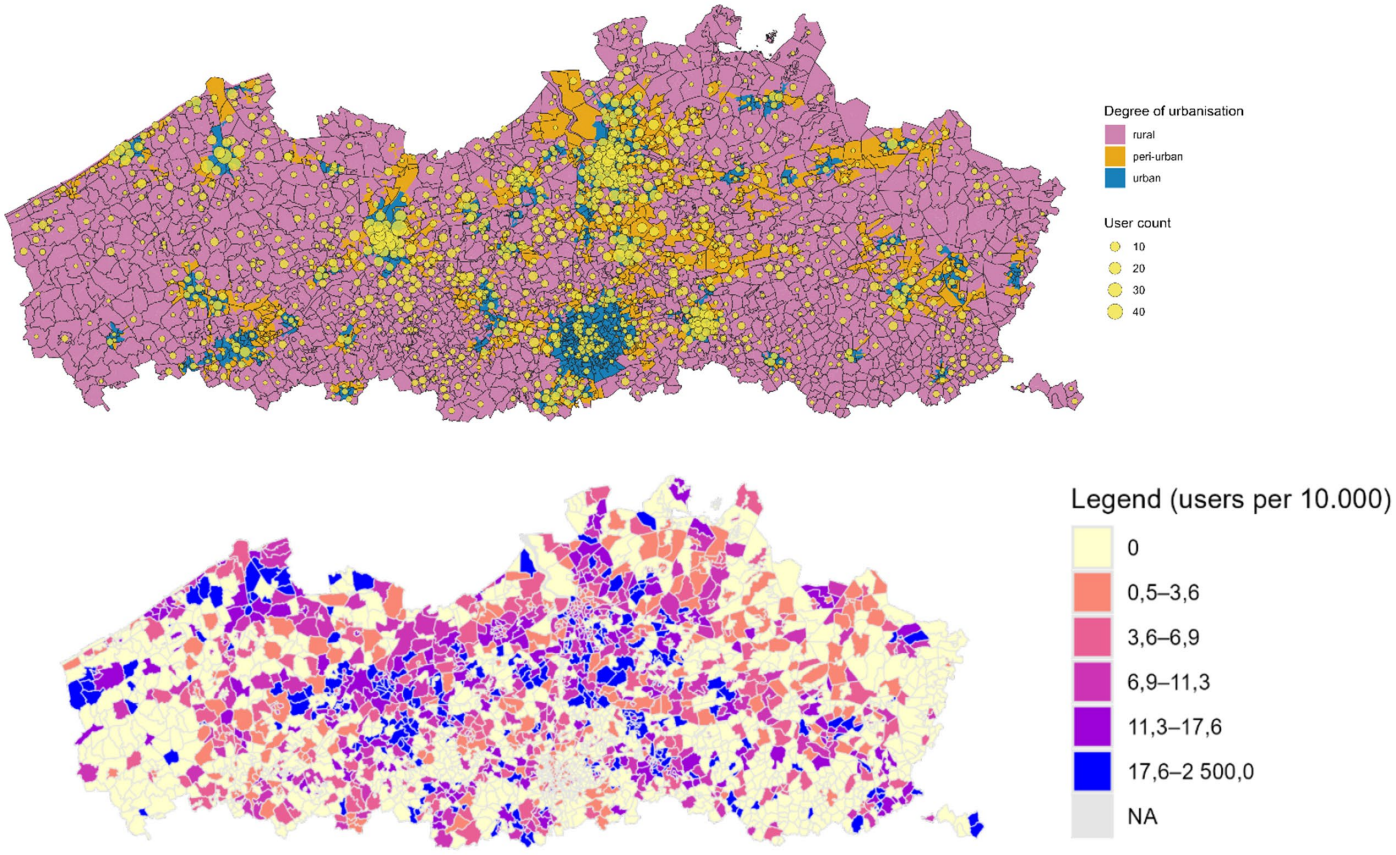
Number of users according to degree of urbanization (upper map) and number of users per 10,000 residents (lower map).

There was a good spread of users over rural (35%), peri-urban (22%) and urban (43%) areas in Flanders (Figure 2).

**Figure 3:**
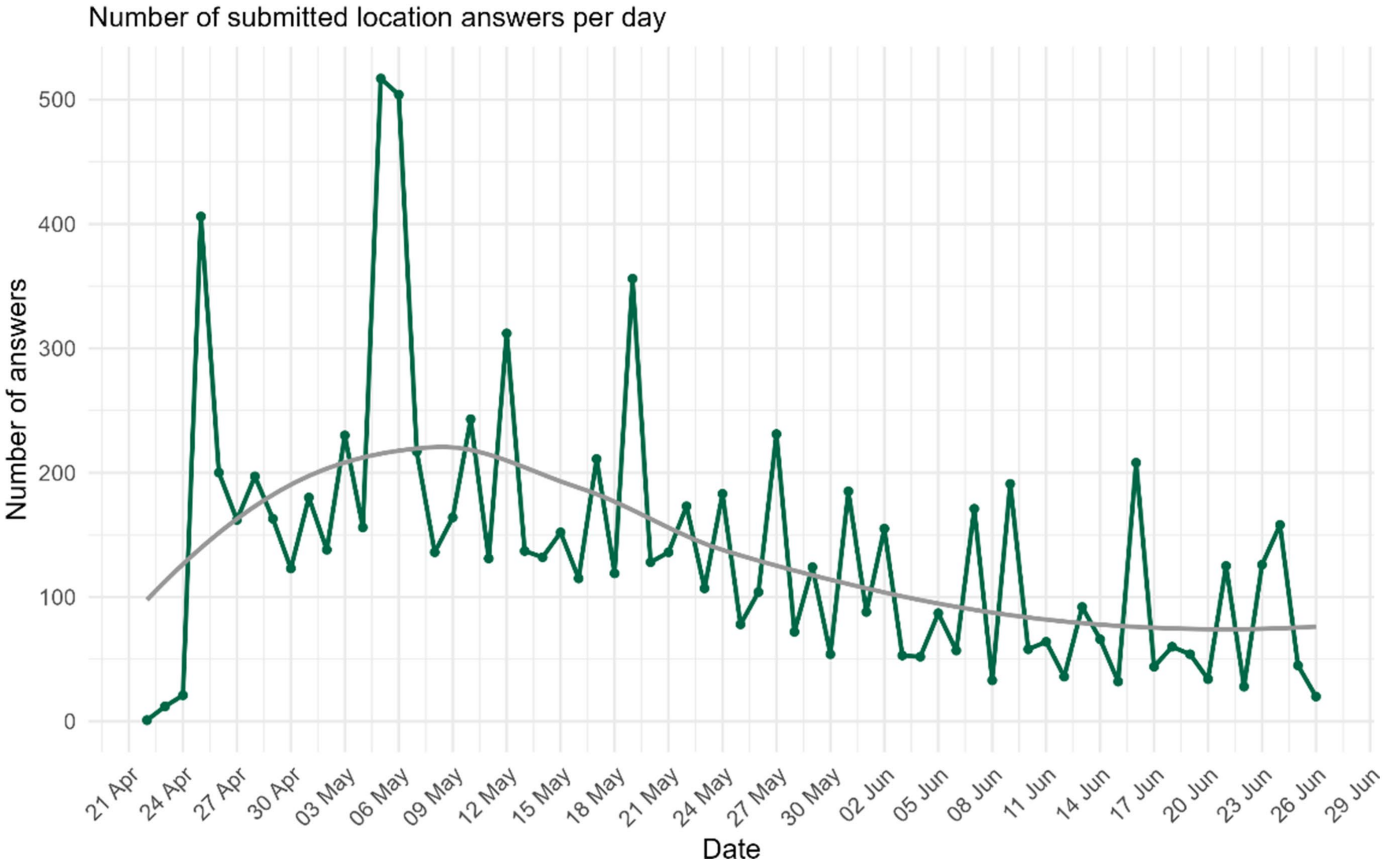
Daily participation (number of submitted location answers per day) over the eight-week campaign of the Big Food Map app.

**Figure 4:**
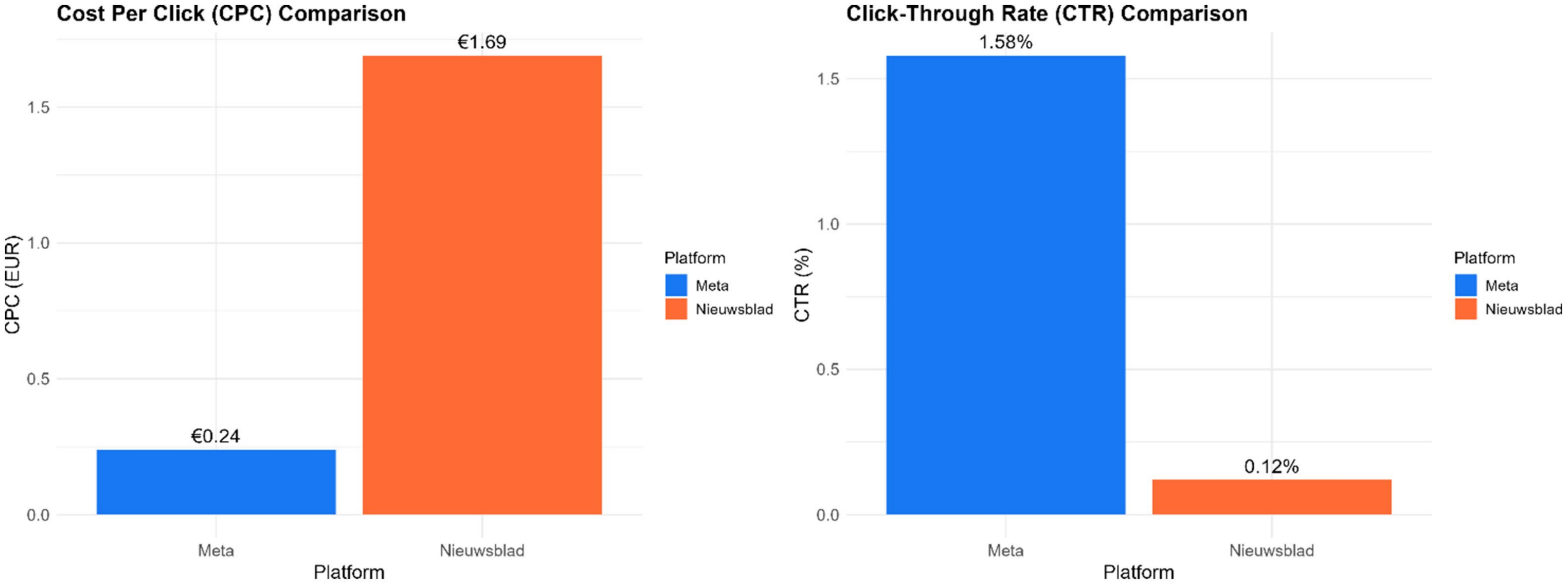
Cost per click and click-through-rate for online newspaper versus meta advertisements.

Half of the registered citizens did not contribute data on weekly challenges, location reviews or daily questions. The use of the app slowly declined over the course of the eight-week campaign and the peaks in use coincided with communication efforts (posts on HOPLR, social media, press release and newspaper articles) (Figure 3). There were 9 HOPLR posts, each viewed between 180,123 and 264,641 views. Meta ads were more cost-effective than the online newspaper ads (Figure 4). Despite wide reach, sustained engagement remained a challenge.

### Evaluation of the app and participatory process

**Figure 5:**
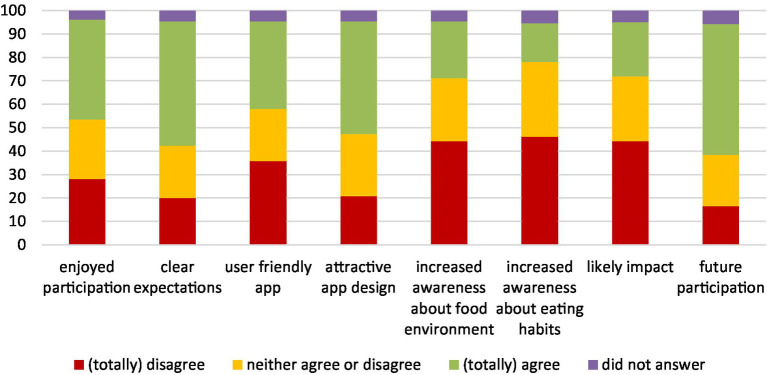
Aspects related to participation, content, and design of the app and possible impact evaluated by citizens on a Likert scale (totally disagree ➔ totally agree).

About 260 Flemish citizens completed an evaluation questionnaire (Figure 5). More than 40% of citizens agreed or totally agreed that participation in the project was an enjoyable experience and 55% of citizens agreed or totally agreed that they would participate again in a future similar project. More than half of citizens agreed or totally agreed that the expectations and goals of the project were clear.

About half of citizens agreed or totally agreed that the design of the app was attractive, whereas less than 40% of citizens agreed or totally agreed that the app was user friendly. In terms of (potential) impact, even if the data collection period occurred during a few months only, almost 25% of citizens agreed or totally agreed that it increased their awareness on food environments, whereas 16% agreed or totally agreed it increased their awareness on their dietary behaviors, and 23% agreed or totally agreed that it is likely to have an impact on local action to improve food environments (Figure 5).

Over the course of the eight-week data collection campaign, 11 messages to engage citizens were posted on the social media platform HOPLR. These messages gathered 463 comments and 2,615 expressions (of which 168 applause emoticons, 84 laughs, 112 heart eyes, 105 likes, 135 sad faces, 1929 thanks and 82 wow emoticons) from citizens. In terms of expressions 95% was positive overall, while 5% was negative (data not shown).

The most common points for improvement identified by citizens across the evaluation questionnaire and HOPLR posts were to provide a web version of the app for citizens without smartphones, providing the options to add food locations (in particular short chain initiatives which are often missing) on the map, more advanced search functions (by food location or option to have the map at current location) for food locations on the map, more options for more contextualized feedback from citizens in regards to the answers or pictures given, more focus on environmental issues, easy removal of questions and challenges not relevant to them or also being able to answer why certain locations are not visited instead of only those that are visited. Some participants found it burdensome to participate or indicated the initiative was not very visible.

A few citizens also did not like the idea of a government-linked research institution gathering data on perceptions and behaviors from them or mentioned they think that eating habits is a matter of individual responsibility only (data not shown).

**Figure 6:**
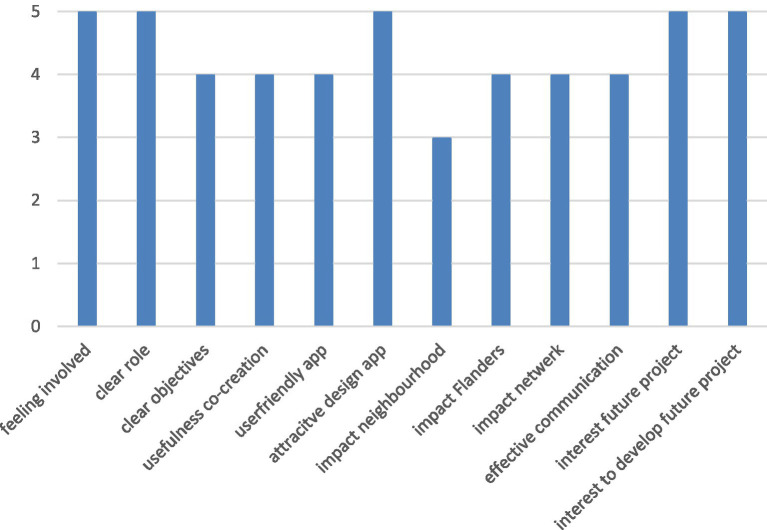
Aspects related to participation, co-creation, and design of the app and possible impact evaluated by partner organizations on a Likert scale–median score (totally disagree ➔ totally agree).

About 7 people across 5 partner institutions filled in the questionnaire – the median scores (across 1/2 (totally) disagree to 4/5 (totally) agree) are presented in Figure 6.

In terms of the co-creation partners highlighted that a more intensive peer-to-peer program with young people would have been beneficial to more actively think about this theme and about their voice. In addition, involving more organizations working with vulnerable groups and more iterations on the testing of the prototype of the app would have been helpful. More impactful communication could have been achieved with a better win-win partnership with the main newspaper involved and some physical marketing in addition to online marketing. Similar comments as those by citizens were given on user friendliness of the app (Figure 6).

#### Context

The development and uptake of the Big Food Map app were shaped by a favorable but also challenging context. On the one hand, the participatory co-creation process ensured that diverse perspectives were represented, including those of adolescents, and adults on lower incomes, as well as local governments, retailers, and short-chain initiatives. This diversity contributed to an inclusive design and a strong sense of legitimacy.

On the other hand, structural barriers such as limited resources, the complexity of food environments, gaps in visibility (e.g., short-chain outlets not always included on the map), and lack of alternatives for citizens without smartphones/internet connection constrained the full reach of the initiative. The communication campaign, while reaching large numbers, also revealed that visibility and sustained engagement required more diversified strategies, including offline approaches and stronger partnerships with media partners.

#### Mechanisms

The app activated several mechanisms that explain how participation translated into engagement. For citizens, the main drivers were ownership, empowerment, and awareness: participants felt that their voices were heard, that they could contribute actively to mapping their environment, and that the process raised their awareness of how surroundings influence eating habits to some extent. For local actors, mechanisms such as legitimacy, shared understanding, and practical usefulness were key. Co-creation workshops and group model building fostered collective insights across stakeholders. Yet, barriers in user-friendliness of the app and in maintaining motivation over time limited the strength of these mechanisms.

#### Outcomes

The outcomes observed were modest but meaningful within the short evaluation period. Among citizens, the app increased perceived awareness of food environments (about a quarter of respondents reported higher awareness), and for more than half it reinforced willingness to participate in similar initiatives in the future. Among stakeholders, the process was valued for generating a comprehensive overview of priority dimensions and settings, and for creating new opportunities for dialog and policy engagement. However, impact on actual behavior change or concrete policy measures remained limited within the project’s timeframe, highlighting the need for longer-term follow-up and structural embedding of such citizen science tools into local governance and policy processes. The next phase of the project aims to co-create concrete actions with stakeholders, especially local governments, retailers and short chain initiatives, based on the results of the study.

## Discussion

### Overview of main results

This study evaluated the Big Food Map, a participatory citizen science initiative in Flanders that combined subjective perceptions and objective geospatial data to stimulate local action on food environments. The co-creation process was inclusive, involving adolescents, adults on lower incomes, and local actors such as local governments, retailers, and short-chain initiatives. The app successfully engaged more than 4,000 citizens in mapping their perceptions on and behaviors within local food environments, generating rich insights into the priority dimensions that shape dietary habits, such as affordability, accessibility, and marketing.

The process evaluation, guided by a Context–Mechanism–Outcome framework, showed that inclusive contexts fostered ownership and awareness, while technical barriers and limited visibility constrained long-term outcomes.

These findings highlight both the promise and the challenges of embedding citizen science approaches into local public health policy and practice. These results align with prior work emphasizing that citizen science can both produce useful data and build community capacity for change (17, 31).

### Key lessons learnt

A first lesson is the value of co-creation in ensuring that the design of digital citizen science tools is relevant and legitimate across diverse groups. This resonates with evidence that participatory methods such as photovoice and group model building can amplify underrepresented voices and foster collective ownership (24, 26). A second lesson is that marketing, affordability, and accessibility remain central levers across both citizen and actor perspectives, confirming international literature on obesogenic food environments (6, 20). A third lesson is that while apps can raise awareness, sustained behavior change and policy impact require longer-term embedding and institutional support, stronger media partnerships, consistent with reviews showing that digital citizen science often delivers incremental rather than transformative change in its early stages (28).

The findings illustrate how context, mechanisms, and outcomes are interlinked. The inclusive co-creation context created legitimacy and ownership, which in turn activated mechanisms of empowerment, awareness, and shared understanding among both citizens and local actors. These mechanisms produced promising early outcomes such as increased awareness, and a willingness to engage in future initiatives. However, contextual barriers such as limited visibility of the project, technical challenges in user-friendliness, and resource constraints weakened the strength of these mechanisms and limited the scope of outcomes. This suggests that the full potential of citizen science tools like the Big Food Map can only be realized when supportive contexts are sustained, mechanisms are nurtured through ongoing engagement and feedback, and outcomes are monitored over longer timeframes.

### Strengths and limitations

The strengths of our approach include the participatory co-design of the digital tool, the mobilization of citizens in the local food-environment context of Flanders, and the combination of objective and subjective data on food environments. The use of a CMO framework further enhanced understanding of how and why outcomes were produced. The current study confirms that citizen science offers a promising route for advancing food-environment research. As discussed in recent reviews, citizen science in food systems has been underutilized but holds particular value for generating localized evidence, engaging citizens as change agents, and bridging research and policy (32). The present project illustrates how a mobile platform can serve as a bridge between citizens, data and decision makers. Hognogi et al. (33) reviewed 303 mobile-app initiatives and concluded that digital tools are becoming foundational in citizen science, enabling monitoring, validation and participatory methods. The Big Food Map app enabled rapid capture of geolocated, photo-validated food-environment features, and the dashboard helped visualize findings for stakeholders.

However, several limitations should be acknowledged. First, while participation was broad, it may not be fully representative of all population sub-groups (e.g., non-smartphone users, vulnerable other than lower-income groups), a common equity challenge in digital health research (21). This also aligns with general citizen science concerns about inclusivity and equity (34). Second, the app faced some technical and usability challenges, which may have discouraged repeated use and impeded sustained engagement. Third, the short data collection period limited assessment of longer-term impacts on behaviors or policy processes. Aylward et al. (17) show that citizen science processes require attention to relationship building, context, turnover, and consensus-building. Finally, the reliance on self-reported perceptions introduces potential reporting biases, though these are partly offset by the triangulation with geospatial data. The current evaluation measures perceived changes in awareness or behavior; multiple waves of the project in the longer term will be needed to measure if awareness and/or behavior actually change.

### Implications for future research and monitoring

In the next phases of the project, key results on perceptions and behaviors will be analyzed, and workshops will be held with the actor groups to define key priorities for action on food environments in Flanders.

Future research should explore how citizen science initiatives like the Big Food Map can be sustained and scaled over time. This includes evaluating how repeated use of the app might influence dietary behaviors, food purchasing patterns, and civic engagement. Linking app-based data with health or consumption outcomes could provide stronger evidence of impact. Longitudinal research could also clarify whether awareness raised through citizen science translates into measurable shifts in food environments or health outcomes, as suggested by early evidence in other fields of participatory public health (19). From a monitoring perspective, the Big Food Map demonstrates the feasibility of collecting granular, neighborhood-level food environment data at scale, which could complement existing INFORMAS monitoring frameworks (6) and be institutionalized as part of local or regional food system surveillance. Future work should explore how citizen-led monitoring can be institutionalized (e.g., through municipal policy frameworks, data sharing platforms) and how it influences decision-making pathways. As computational tools proliferate, future projects could incorporate machine-learning image-recognition of food-outlets, automated classification of healthy/unhealthy promotions, and real-time dashboards to support decision-making (35).

### Implications for actions and policy

For local governments, the Big Food Map offers a promising tool to identify priority settings and dimensions where interventions can be most effective, such as reducing the visibility of unhealthy food marketing, improving affordability of fresh produce, and ensuring accessibility of healthy outlets. The app also fosters dialog between municipalities and citizens, which could serve as a catalyst for more integrated food strategies at the local level.

However, structural support is needed to overcome limitations identified in the process evaluation — for example, ensuring visibility of short-chain initiatives, providing web-based alternatives for digitally excluded groups, and embedding citizen science outputs into municipal decision-making processes. Ultimately, citizen-driven tools such as the Big Food Map can help bridge the gap between community insights and structural policy actions, provided that governments are willing to integrate them into decision-making processes.

One of the key aspirations of food-environment monitoring is to influence policy and practice — moving beyond data generation to action. The transition from monitoring to action is as important as data collection. In the context of food-environment policy, where regulations (e.g., zoning, advertising restrictions) are often under-utilized, citizen-led data may help address accountability and transparency gaps.

## Conclusion

This study contributes to the growing evidence base that citizen science, when supported by digital tools and stakeholder linkages, can enable meaningful monitoring of food-environments. The “Big Food Map” app exemplifies how co-design, mobile mapping and reflection workshops can foster empowerment and awareness among citizens and local actors and generate actionable insights. Ensuring sustained visibility, technical usability, and structural embedding into local governance will be essential to harness the full potential of such initiatives in creating healthier food environments. In addition, monitoring alone is not sufficient; translating data into sustained policy or practice change requires embedding tools into governance structures, addressing representativeness, and leveraging digital analytics. As food-environment challenges grow and policy opportunities remain under-realized, participatory digital monitoring offers a promising pathway, but one that must be intentionally designed, inclusive, and linked with systems of decision-making to deliver impact.

## Data Availability

The raw data supporting the conclusions of this article are available from the authors upon reasonable request.
