# Supplementary material for: Citizen science for healthier food environments: a realist-informed process evaluation of the big food map app in Flanders, Belgium
Source: Front Public Health. 2026 Mar 10;14:1742139. doi: 10.3389/fpubh.2026.1742139 (PMC13008956; doi:10.3389/fpubh.2026.1742139)
Supplement: Supplementary file 3 [file Table_3.DOCX]

Annex 3 - branding

**© De Grote Voedselkaart by Leap Forward**

**Icons**

**
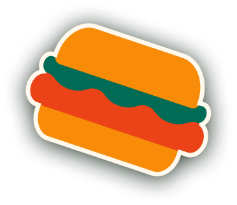

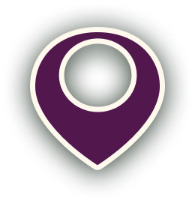

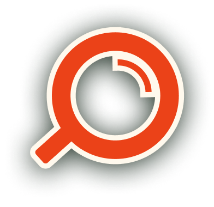

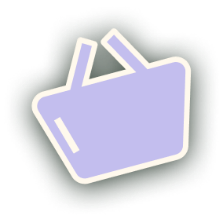

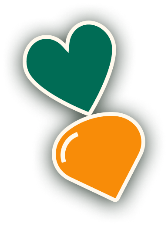
**

**
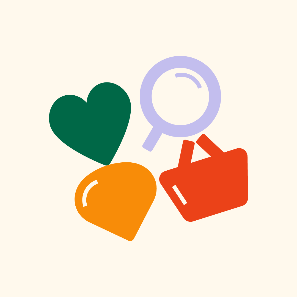

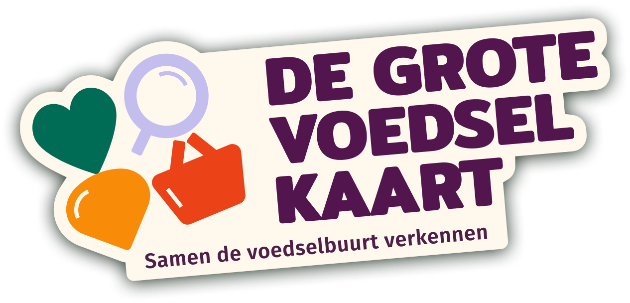

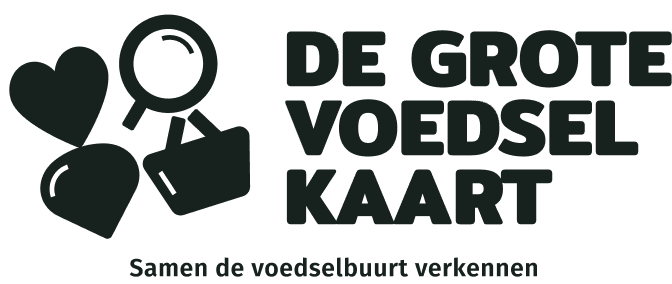

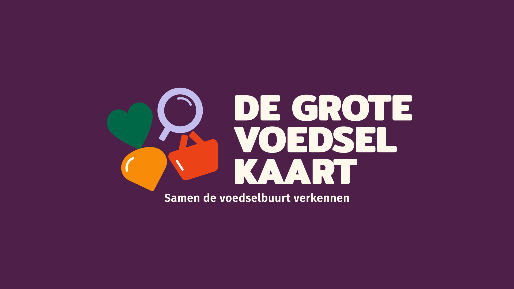
Logos**

**Frames**


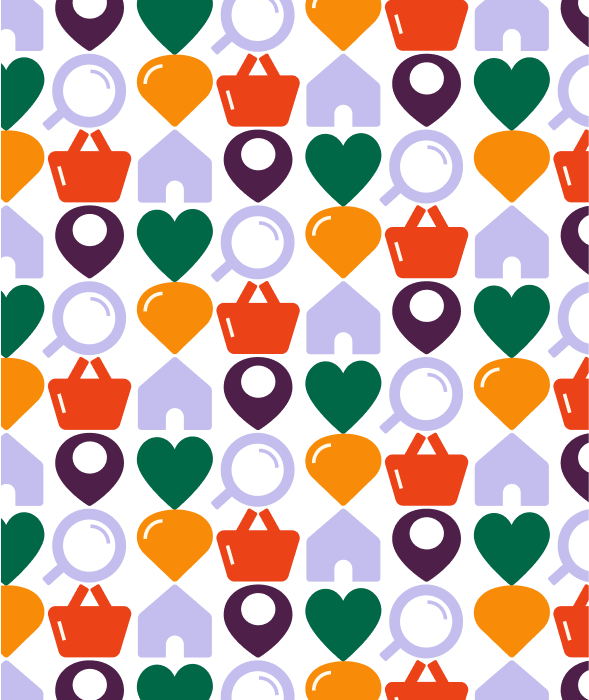

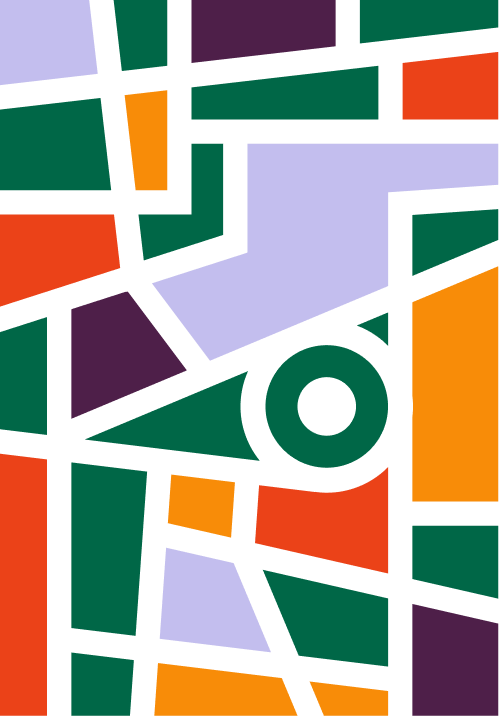

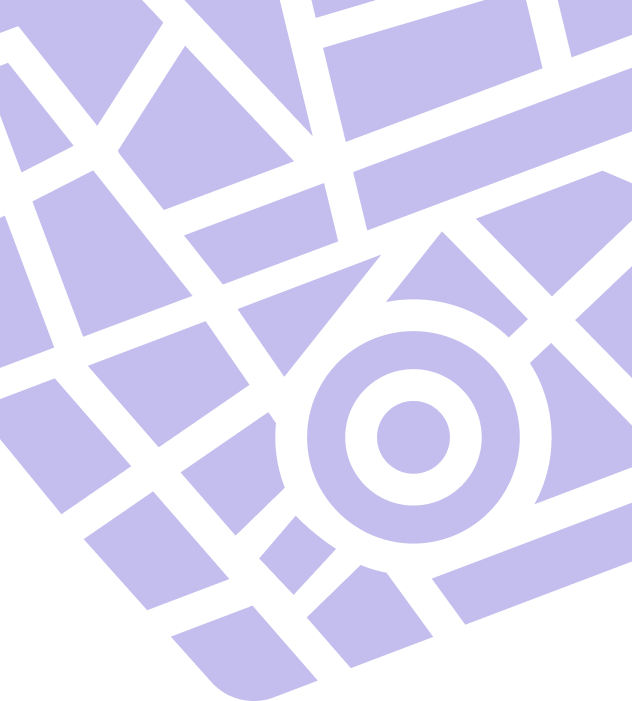


**
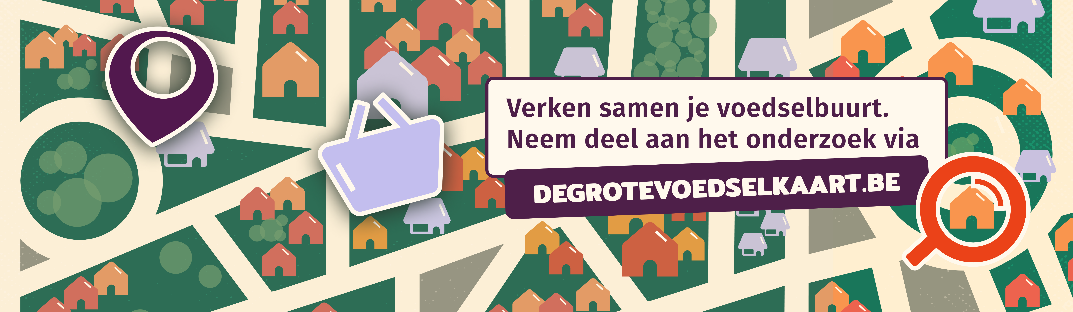

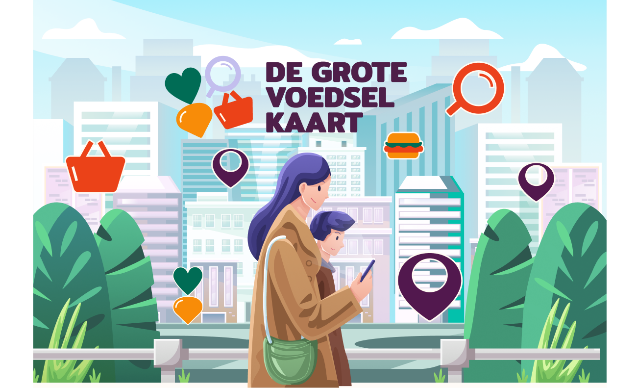

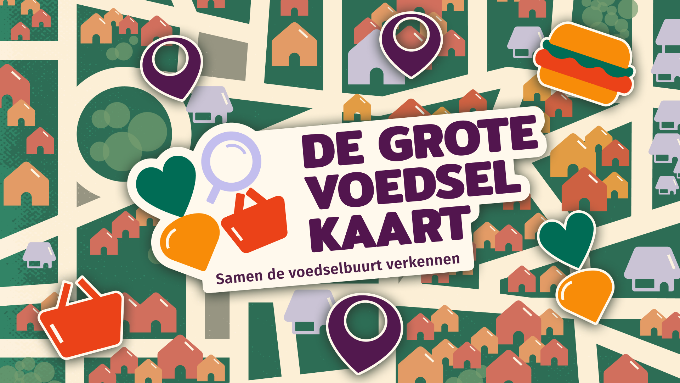
Headers and banners**

**App design**

**
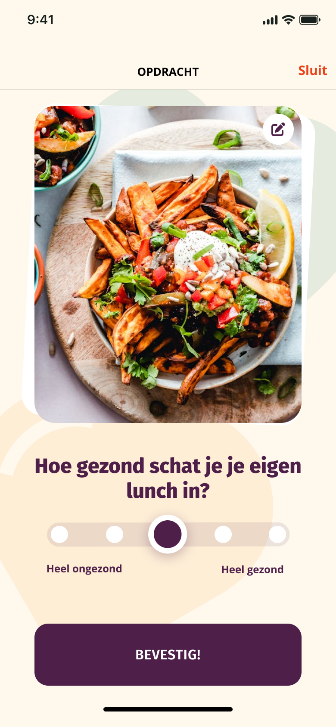

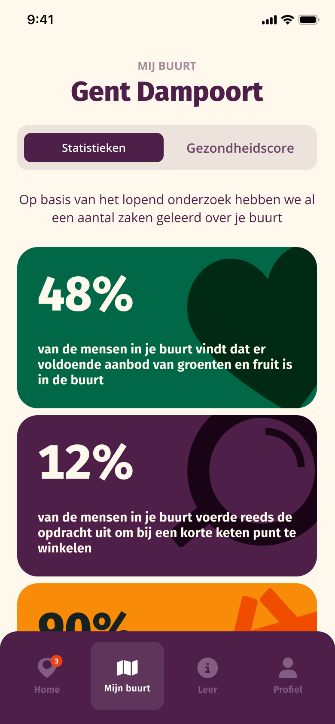

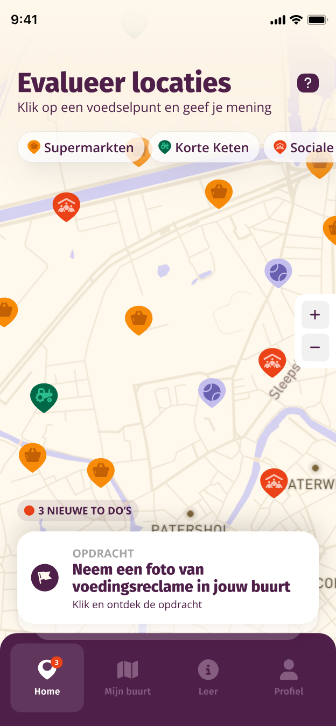

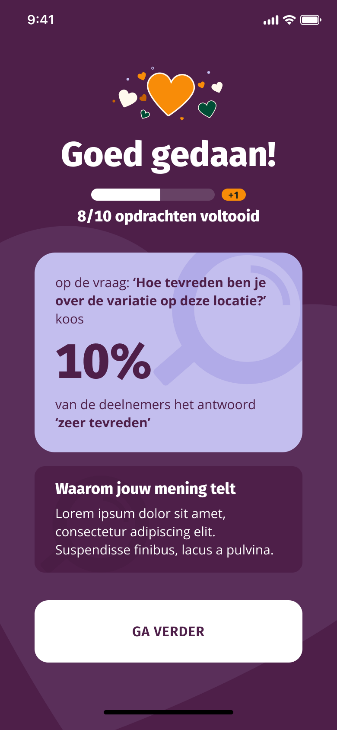
**
